# Identifying substance misuse related foetal anomaly cases in routinely collected data

**DOI:** 10.23889/ijpds.v9i5.2789

**Published:** 2024-09-18

**Authors:** Ian Farr, Hywel Evans, Columbus Ohaeri, Ryan Phillips, Olabambo Oluwasuji, Josh Dixon, Grace Bailey, Josie Smith

**Affiliations:** 1 Swansea University; 2 Public Health Wales; 3 Welsh Government

## Objective

Foetal Alcohol Syndrome Disorder (FASD) and substance misuse (SM) related foetal anomalies are among the most severe consequences of SM during pregnancy. These are preventable lifelong developmental disorders that affect children’s health, education, and future prospects. Despite the adverse effects of these disorders, epidemiological research in this population remains challenging due to clinical and administrative underreporting. For the first time, SM-related foetal anomaly prevalence in Wales are reported using linked routine data.

## Approach

We combined birth register data with primary and secondary healthcare records to identify infants diagnosed with SM-related foetal anomaly. To compare, we identified mothers referred for SM treatment during pregnancy. The frequency and type of maternal SM-related healthcare interactions preceding birth (within three years prior) were described.

## Conclusions

A novel method to identify SM-related foetal anomaly cases using routinely collected data in Wales is provided. The published code to identify mothers and children exposed to SM-related events can also be reused in future projects. When compared with published estimates, cases of SM-related foetal anomaly derived from primary and secondary care diagnoses in Wales are likely underreported, along with the scale of the harm.

## Implications

We highlight an unmet need, by counting mothers who were not in SM treatment but had SM-related health events in Wales before giving birth. By further understanding of the risks of SM on unborn children, this body of work provides insight to develop policy and clinical interventions to help future generations. We advocate introducing a SM foetal anomaly database for the UK.

